# 2409. Potential candidates for partial oral treatment in patients with endocarditis at a US center

**DOI:** 10.1093/ofid/ofad500.2029

**Published:** 2023-11-27

**Authors:** Mia M Pries-Heje, Andy J Kim, Mary-Ruth M Joyce, Dylan J Hartman, Erin M Connolly, Akinobu Itoh, Mark Cunningham, Patrick O’Gara, Yee-Ping Sun, James Maguire, Ayaz Aghayev, Brittany N Weber, Marcelo Di Carli, Joji Suzuki, Yesenia Buenrostro Ramirez, Kasper K Iversen, Lindsey R Baden, Henning Bundgaard, Ann E Woolley

**Affiliations:** Rigshospitalet, University of Copenhagen, Copenhagen, Hovedstaden, Denmark; Brigham and Women's Hospital, Boston, Massachusetts; Brigham and Women's Hospital, Boston, Massachusetts; Brigham and Women's Hospital, Boston, Massachusetts; Brigham and Women's Hospital, Boston, Massachusetts; Brigham and Women's Hospital, Boston, Massachusetts; Brigham and Women's Hospital, Boston, Massachusetts; Brigham and Women's Hospital, Boston, Massachusetts; Brigham and Women's Hospital, Boston, Massachusetts; Brigham and Women’s Hospital and Harvard Medical School, Boston, Massachusetts; Brigham and Women's Hospital, Boston, Massachusetts; Brigham and Women's Hospital, Boston, Massachusetts; Brigham and Women's Hospital, Boston, Massachusetts; Brigham and Women's Hospital / Harvard Medical School, Boston, MA; Brigham and Women's Hospital, Boston, Massachusetts; Herlev-Gentofte University Hospital, University of Copenhagen, Copenhagen, Hovedstaden, Denmark; Brigham and Women's Hospital, Boston, Massachusetts; Rigshospitalet, University of Copenhagen, Copenhagen, Hovedstaden, Denmark; Brigham and Women's Hospital, Boston, Massachusetts

## Abstract

**Background:**

In the Danish POET trial, oral step-down oral antibiotics in patients with left-sided infective endocarditis (IE) was non-inferior to traditional intravenous antibiotics (IV-AB). Outpatient parenteral antimicrobial therapy (OPAT) is widely used in the US despite associated adverse events. Our aim was to assess the proportion of US patients that were potential candidates for step-down oral antibiotic treatment, i.e. POET-treatment, and number of days IV-AB antibiotics could have been reduced.

**Methods:**

Retrospective study of patients admitted with left-sided IE at a US single center from January 2021 – June 2022 was conducted. Patients were considered candidates for POET-treatment if they were infected with *S. aureus, E. faecalis, Streptococcus* spp., or CoNS, had a BMI < 40 kg/m^2^ and survived until discharge. The primary outcome was a composite of all-cause mortality, symptomatic embolism, unplanned cardiac surgery, and relapse of bacteremia within 6 months.

**Results:**

Out of 111 patients with left-sided IE, 71 (64%) were considered potential POET-candidates (median age 64 years (IQR 54-75), 72% male); 30% had IE caused by *S. aureus*, and 48% were surgically treated (Table 1). Potential candidates were older (64 vs 60 years), more often infected with Streptococcus spp. (24% vs 0%) or CoNS (23% vs 5%), and had a pacemaker/ICD at baseline (23% vs 5%), as compared to patients who were not candidates for POET-treatment. Of potential POET-candidates, 93% (66/71) were stabilized, allowing discharge to OPAT with a median of 29 days (IQR 20-36) of antibiotic treatment remaining.

The primary outcome occurred in 28% of potential POET-candidates and in 53% of non-POET candidates, compared to the occurrence of the primary outcome in 11% in the Danish POET-trial (Table 2).

**Figure:**
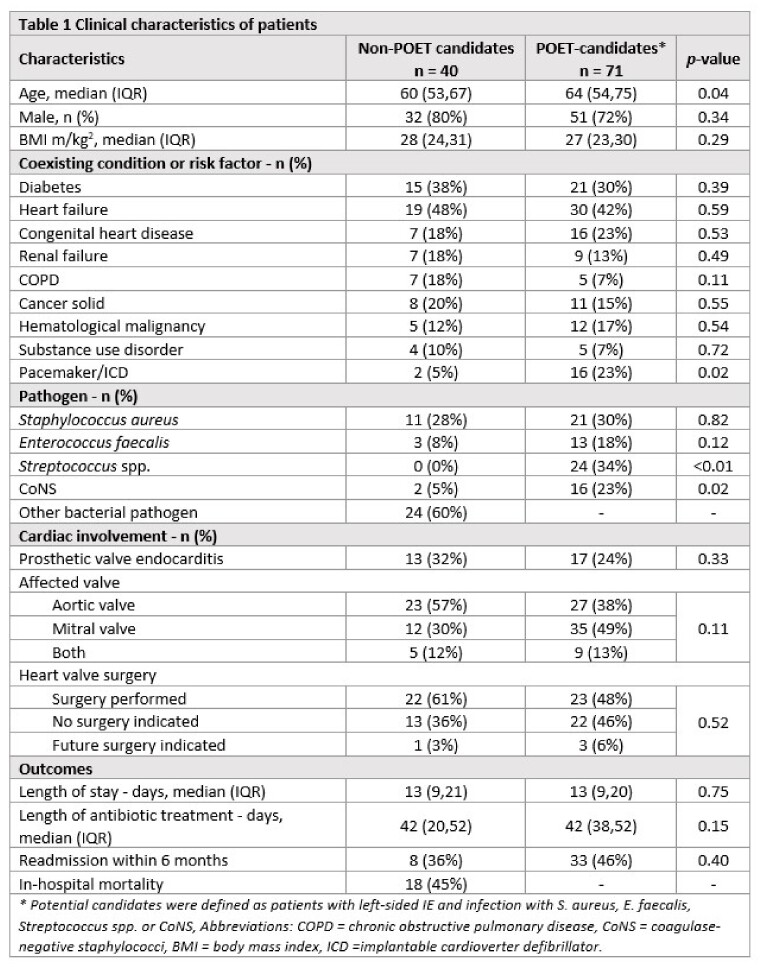

**Figure:**
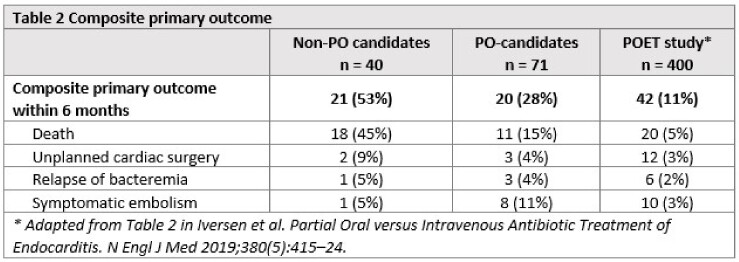

**Conclusion:**

More than half of patients admitted with left-sided IE were potential candidates for POET-treatment. For the vast majority of these patients, up to 4 weeks of IV-AB may have converted to oral step-down antibiotics. Prevalence of the primary outcome in potential POET-candidates was almost 3 times higher in this US cohort than in the Danish POET trial, indicating a higher baseline risk. These findings indicate a need for prospective studies of oral step-down antibiotic therapy in US patients with IE.

**Disclosures:**

**Joji Suzuki, MD**, Indivior: Grant/Research Support

